# A Rare Case of Subacute Painful Thyroiditis Causing Thyroid Storm and a Successful Trial of Propylthiouracil

**DOI:** 10.7759/cureus.9461

**Published:** 2020-07-29

**Authors:** Salem Gaballa, Kyaw M Hlaing, Nadine Bos, Safa Moursy, Mustafa Hakami

**Affiliations:** 1 Internal Medicine, LewisGale Medical Center, Salem, USA; 2 Internal Medicine, Lewisgale Medical Center, Salem, USA; 3 Psychiatry, Lewisgale Medical Center, Salem, USA

**Keywords:** subacute granulomatous thyroiditis, de quervain thyroiditis, thyroid storm, propylthiouracil (ptu)

## Abstract

Thyroid storm is a rare, life-threatening condition characterized by severe or exaggerated clinical manifestations of thyrotoxicosis, commonly occurring in patients with longstanding, untreated hyperthyroidism such as Graves' disease and toxic nodular goiter. Subacute painful thyroiditis, also known as de Quervain thyroiditis, is a self-limited inflammatory disease of the thyroid gland that is characterized by neck pain, a tender diffuse thyroid goiter, elevated inflammatory markers, and a predictable course of thyroid function evolution. Rarely, it can cause thyroid storm. Herein, we report a rare case of a 25-year-old woman who was admitted for sepsis and acute painful thyroiditis who then developed thyroid storm. The patient was treated in the intensive care unit (ICU) and responded very well to steroids, propranolol, a seven-day trial of propylthiouracil, and ultimately achieved a euthyroid state on discharge.

## Introduction

Subacute thyroiditis (SAT) has other names, including subacute granulomatous thyroiditis, painful thyroiditis, and de Quervain’s thyroiditis. Subacute thyroiditis is an uncommon etiology of hyperthyroidism or thyroid storm and affects women more often than men (3 to 5:1 ratio) [1]. It has a typical presentation of neck pain, diffuse tender goiter, and a predictable course of thyroid function tests. Usually, it presents with hyperthyroidism followed by euthyroidism, hypothyroidism, and, subsequently, recovery of normal thyroid function [2]. The reported incidence of the SAT is 12.1 cases per 100,000/year, with a higher incidence in females than in males (19.1 and 4.1 per 100,000/year, respectively) [3].

## Case presentation

A 31-year-old Caucasian female with a past medical history significant for intravenous (IV) drug abuse, hypertension, and bipolar disorder was brought to the emergency department with complaints of shortness of breath, palpitations, and neck pain. The patient endorsed associated pleuritic chest pain, generalized weakness, fatigue, unintentional 10-pound weight loss, profuse sweating, diffuse abdominal pain, and nausea. She also endorsed recent flu-like symptoms, which resolved after a few days. She denied any heat or cold intolerance, tremors, and bowel habit changes. She endorsed dysuria but denied hematuria or urgency. She had a 15 pack-year smoking history, drank alcohol occasionally, and had been sober from IV drug abuse for four years. She denied any family history of thyroid disease or thyroid cancer.

Physical examination was remarkable for tachycardia (without murmurs), a tender diffuse goiter without any bruit or lymphadenopathy, dry mucosal membranes, and tachypnea without any abnormal respiratory sounds. Vital signs were remarkable for temperature (T) 99.2 F°, heart rate (HR) of 138, and respiratory rate (RR) of 22.

Labatory findings (Table 1) revealed the following: white blood cells (WBCs) 9.26 cells/mcL, hemoglobin 13.8 g/dL, and platelets 155 cells/mcL. Basic metabolic profile (BMP) revealed sodium (Na) 126 mEq/L, potassium (K) 3.2 mEq/L, chloride (Cl) 102 mEq/L, carbon dioxide (CO_2_) 28 mEq/L, blood urea nitrogen (BUN) 14 mg/dL, creatinine 0.8 mg/dL, and albumin 1.6 g/dL. The liver function panel was within normal limits. Troponins were <0.015 ng/mL, erythrocyte sedimentation rate (ESR) was 91 mm/h, and C-reactive protein (CRP) was 19 mg/L. Procalcitonin was 4.9 ng/mL.

**Table 1:** Laboratory findings in the presentation Na, sodium; K, potassium; Cl, chloride; CO_2_, carbon dioxide; BUN, blood urea nitrogen; ESR, erythrocyte sedimentation rate; CRP, C-reactive protein; TSH, thyroid-stimulating hormone; Free T4, free thyroxine; Free T3, free triiodothyronine; TSI, thyroid-stimulating immunoglobulin; TPO antibodies, Thyroid peroxidase antibodies

FreeTests	Result	Reference Range
Hemoglobin	13.8 g/dL	14-16 g/dL
Hematocrit	41.2 %	40-52 %
White cell count	9.26 x 10^9^/L	4-10 x 10^9^/L
Platelet count	155 x 10^9^/L	150-400 x 10^9^/L
Na	126 mEq/L	135-145 mEq/L
K	3.2 mEq/L	3.5-5.2 mEq/L
Cl	102 mEq/L	96-106 mEq/L
CO2	28 mEq/L	23-29 mEq/L
BUN	14 mg/dL	6-20 mg/dL
Creatinine	0.8 mg/dL	0.8-1.2 mg/dL
Albumin	1.6 g/dL	3.4 to 5.4 g/dL
ESR	91 mm/hr	0-26 mm/hr
CRP	19 mg/L	0- 10 mg/L
Procalcitonin	4.9 ng/mL	0.10 - 0.25 ng/mL
Troponin	<0.015 ng/mL	0-0.045 ng/mL
TSH	0.08 μU/mL	0.4-5 μU/mL
Free T4	5.57 ng/dL	0.8-2.8 ng/dL
Total T3	201 ng/dL	60 to 180 ng/dL
TSI	<0.01 IU/L	0-0.5 IU/L
TPO antibodies	<6 IU/mL	0-9 IU/mL

**Figure 1:**
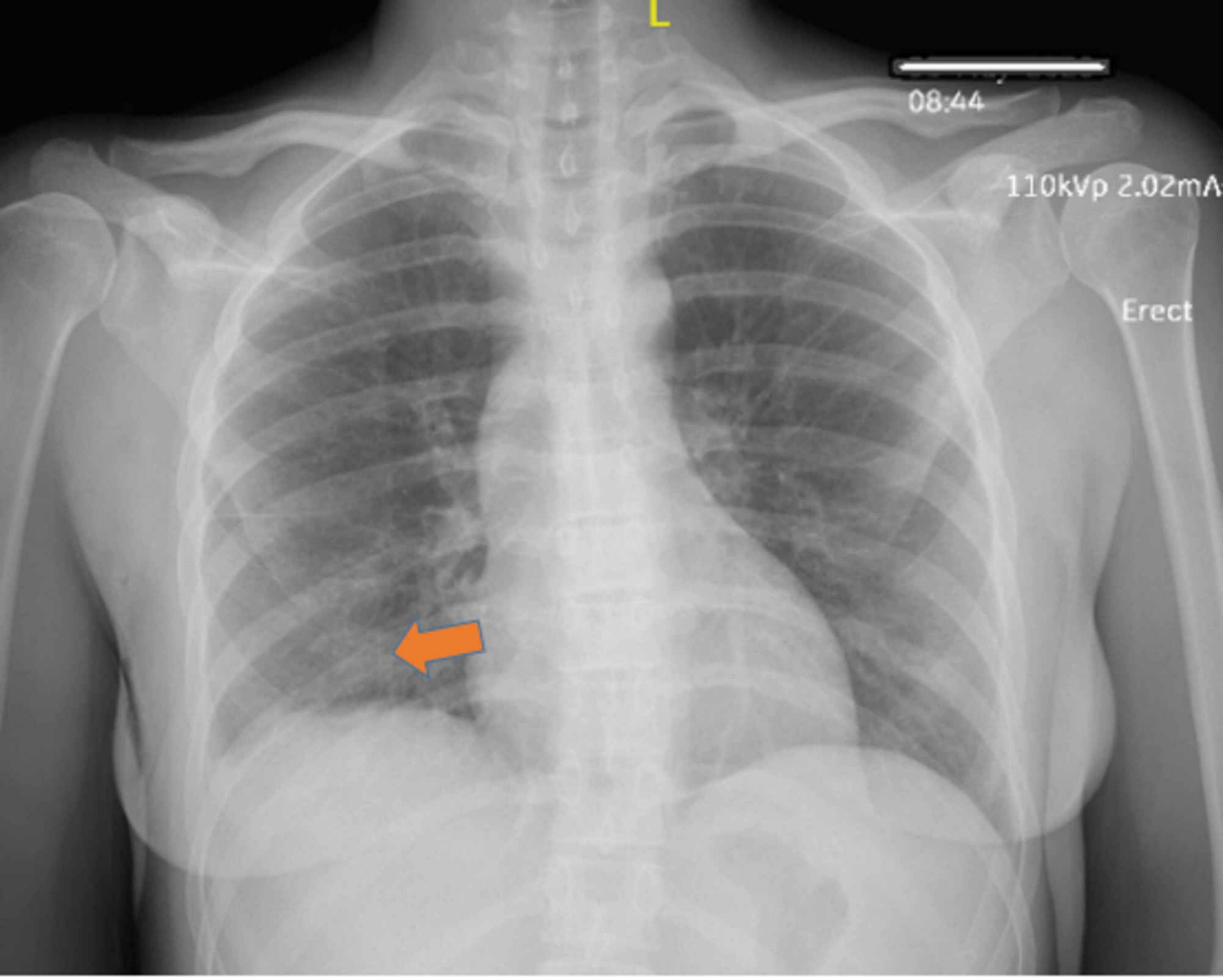
Chest X-ray in anterior-posterior position (AP) showing right lower lobe infiltrate as pointed by the orange arrow

**Figure 2:**
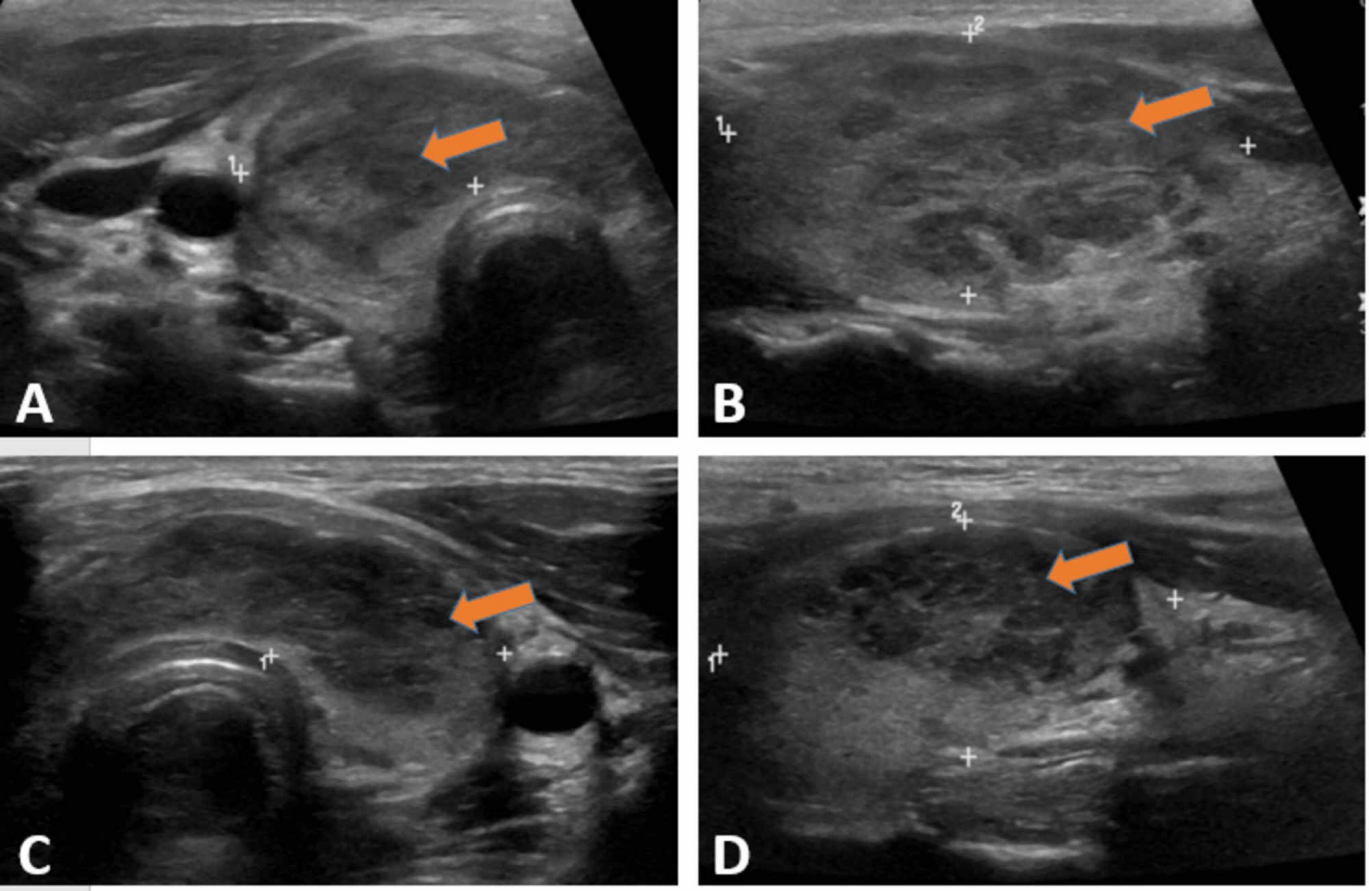
Thyroid ultrasound (US) showing heterogeneous diffuse goiter without any nodules, cysts, or abscess A) Right thyroid lobe in a longitudinal view (48×24×21 mm); B) Right thyroid lobe in a sagittal view; C) Left thyroid lobe in a longitudinal view (40×19×16 mm); D) Left thyroid lobe in a sagittal view

**Figure 3:**
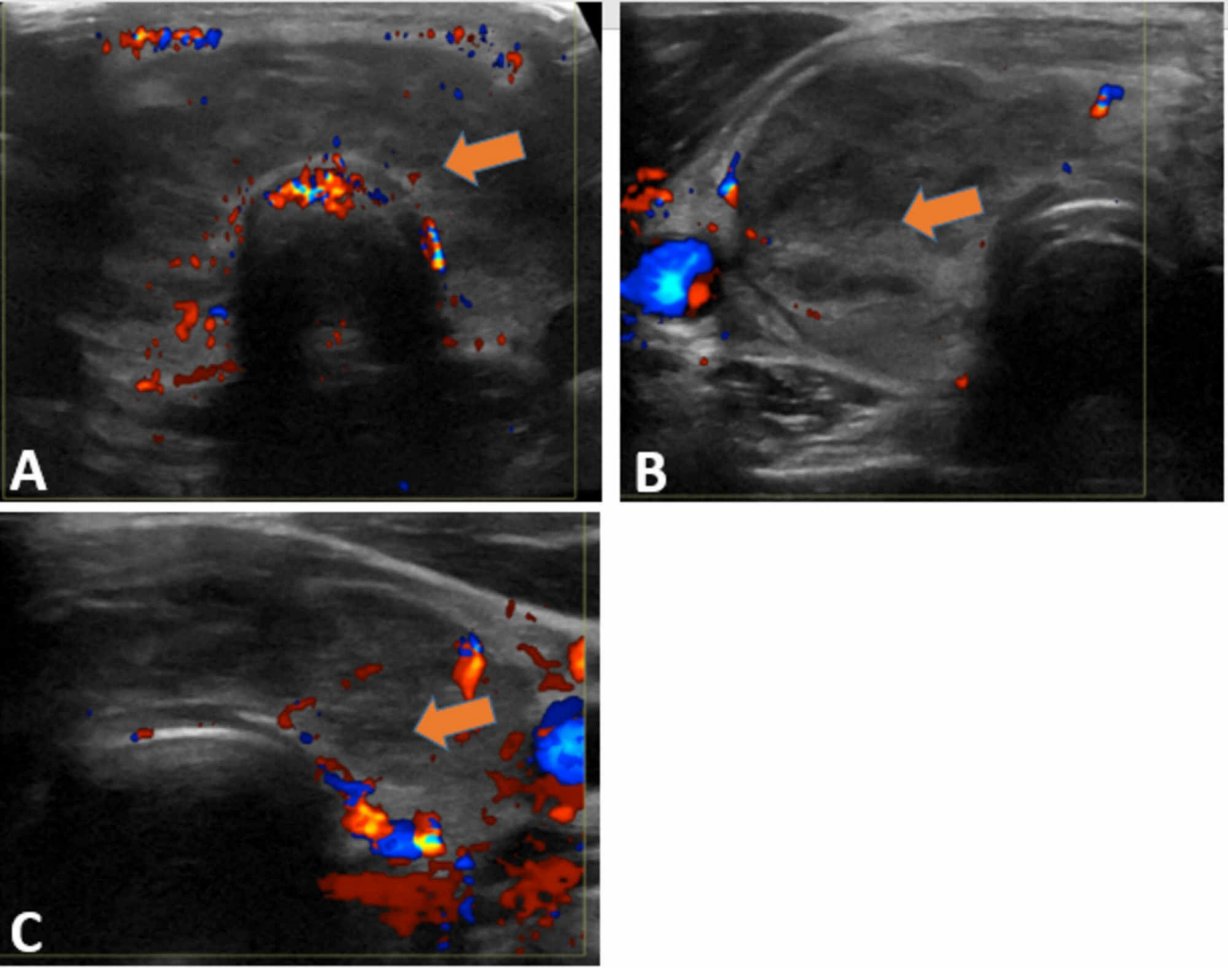
Thyroid Doppler ultrasound (US) showing hypo-vascular diffuse goiter, making the diagnosis of subacute thyroiditis more likely A) Thyroid isthmus, B) Right thyroid lobe, and C) Left thyroid lobe

Electrocardiogram (ECG) showed sinus tachycardia without ST-T wave changes. Chest X-ray (CXR) showed a right lower lobe infiltrate without pleural effusion or reactive lymphadenopathy (Figure 1). Urinalysis revealed positive leukocyte esterase, nitrite, and white blood cells >10. Urine culture was negative. Blood cultures were positive for Escherichia coli (E. coli). Thyroid panel showed a thyroid-stimulating hormone (TSH) of 0.08 μU/mL (normal range 0.4-5 μU/mL), free thyroxine (T4) of 5.57 ng/dL (normal range: 0.8-2.8 ng/dL), total triiodothyronine (T3) of 201 ng/dL (normal range: 60 to 180 ng/dL), thyroid-stimulating immunoglobulin (TSI) <0.01 IU/L (normal range: 0-0.5 IU/L), and thyroid peroxidase antibodies (TPO) <6 IU/mL (normal range < 9 IU/mL). Thyroid ultrasonography (US) revealed heterogeneous diffuse goiter without any nodules, cysts, or abscess (Figure 2). Doppler US revealed a hypovascular thyroid goiter (Figure 3), thus confirming the diagnosis of subacute painful thyroiditis.

**Figure 4:**
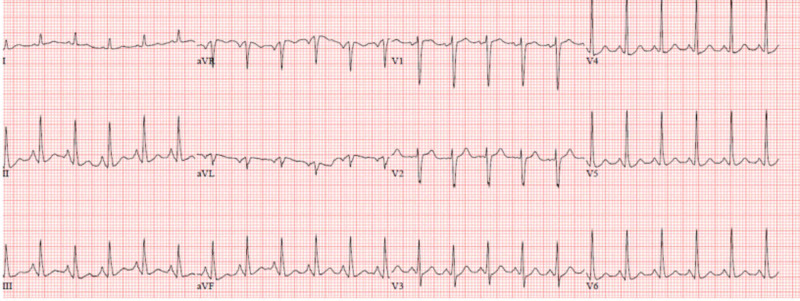
Electrocardiogram showing sinus tachycardia, normal intervals and axis, and without ST-T wave changes

The patient was admitted to the progressive care unit for sepsis secondary to community-acquired pneumonia, E. coli bacteremia, and subacute painful thyroiditis. She was treated with IV fluids (initially 30 cc/kg bolus then 100 cc/hr maintenance), antibiotics, acetaminophen, and propranolol 20 mg every six hours. The second day, the patient triggered a rapid response due to altered mental status, high fever, profuse sweating, and palpitations. Vital signs showed a temperature of 104.5 F°, heart rate (HR) of 138, blood pressure (BP) 110/60, with 100% oxygen saturation on room air. Electrocardiogram (ECG) showed sinus tachycardia without ST-T wave changes (Figure 4). Repeated troponin, lactic acid, and basic metabolic profile were unremarkable. Repeated free thyroxine (FT4) was >8, and thyroid-stimulating hormone (TSH) was undetectable. The patient was transferred to the intensive care unit (ICU) for further management of the thyroid storm. Endocrinology was consulted and recommended cold IV fluids, cooling blankets, acetaminophen 650 mg every six hours, titration of propranolol to 40 mg every six hours, with a target HR <100, hydrocortisone 100 mg once then 50 mg every six hours, and one cholestyramine packet every eight hours. On the third day, repeat FT4 was still >8, with slight clinical improvement of mental status. Endocrinology recommended starting a trial of propylthiouracil (PTU) of 150 mg every eight hours with FT4 daily. Over the next few days, the patient improved significantly and FT4 started to trend down (>8, >8, 6.46, 4.75, 3.48, 2.88,1.58, and 1.33). The patient was transferred out of the ICU, and propranolol, corticosteroids, cholestyramine, and PTU were tapered off over the next few days before she was discharged home. On her follow-up at the endocrinology clinic, her symptoms completely resolved. Repeat thyroid function tests were within normal limits.

## Discussion

**Figure 5:**
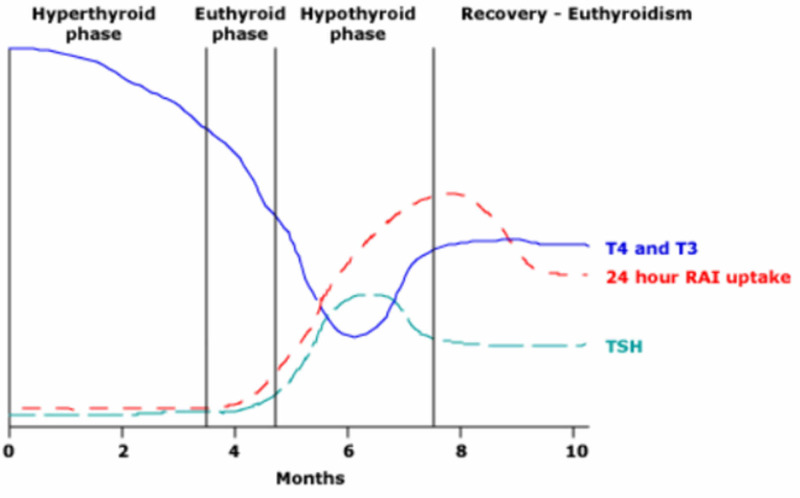
The course of subacute thyroiditis The initial thyroid inflammation damages thyroid follicles and activates the proteolysis of the thyroglobulin stored within the follicles. The result is the unregulated release of large amounts of T4 and T3 into the circulation and, therefore, hyperthyroidism. This state lasts only until the stores of thyroglobulin are exhausted because new hormone synthesis ceases. As the inflammation subsides, the thyroid follicles regenerate, and thyroid hormone synthesis and secretion resume. There may be a transient period of hypothyroidism and increased TSH secretion before thyroid secretion becomes normal again. However, some patients have only a hyperthyroid or hypothyroid phase. Image reproduction approved by Wolters Kluwer. T4: thyroxine; T3: triiodothyronine; RAI: radioiodine; TSH: thyroid-stimulating hormone

Per Desailloud et al., viral infections are frequently cited as a major environmental factor involved in subacute thyroiditis and other autoimmune thyroid diseases [4]. A review by Ohsako et al. concluded that there are at least two types of SAT that can be classified by association with either HLA-B35 or HLA-B67 antigens [5]. It is thought that the disorder stems from a subclinical viral infection that provides an antigen that binds to HLA-B35 molecules on host macrophages. This results in an antigen-HLA-B35 complex, which activates cytotoxic T lymphocytes causing thyroid follicular cell destruction due to structural similarity with the infection-related antigen [6]. The destruction of thyroid follicular cells results in the release of large amounts of T4 and T3 into the circulation, causing clinical and biochemical manifestations of hyperthyroidism. As the inflammatory process subsides, the thyroid follicles regenerate, and thyroid hormone synthesis and secretion recover as shown in Figure 5 [7].

**Figure 6:**
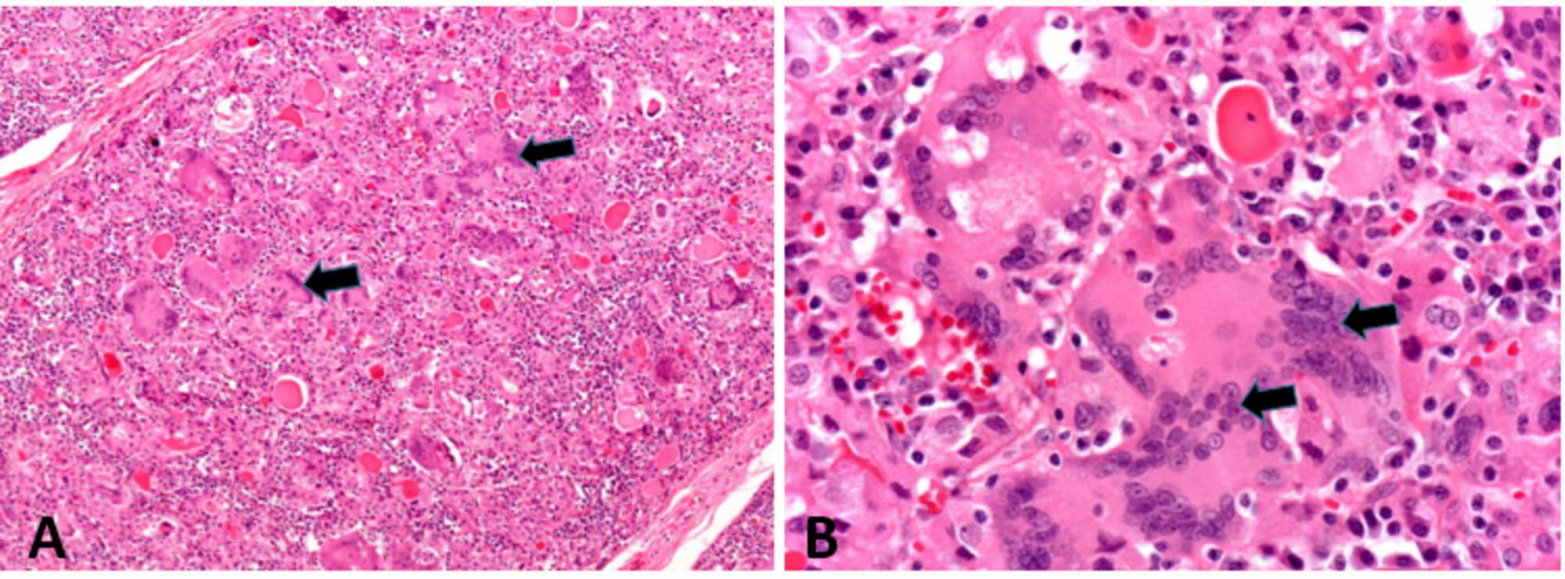
Histopathology of subacute thyroiditis A) Thyroid tissue infiltration with neutrophils, lymphocytes, histiocytes and giant cells, masses of colloid, and necrosis of thyroid follicular cells as pointed by the black arrow; B) on higher magnification showing numerous foreign body giant cells as pointed by the black arrow Courtesy of Dr. Dharam Ramnani [8]

Although the diagnosis is usually clinical, a fine-needle biopsy may be needed to rule out abscess formation. The pathological changes (Figure 6) often reveal widespread infiltration with neutrophils, lymphocytes, histiocytes, and giant cells, masses of colloid, disruption and collapse of thyroid follicles, and necrosis of thyroid follicular cells. Later, there may be some fibrosis, but eventually, the gland histology returns to normal [8].

The clinical manifestations of SAT include tender diffuse goiter and neck pain that usually radiates to the jaw and ears and is exacerbated by coughing or turning the head. Fever, sore throat, generalized weakness, and myalgia are common as well [9]. Generally, SAT and hyperthyroidism are transient and self-limited with improvement in two to eight weeks even if the patient is not treated. Although hyperthyroidism is usually mild and transient, it may rarely cause thyroid storm [10].

In most cases, SAT is a clinical diagnosis, and the presence of neck pain and tender diffuse goiter are sufficient to establish the diagnosis. In addition to the clinical signs of hyperthyroidism, serum TSH is usually suppressed (typically <0.1 mU/L) and free T4 and T3 concentrations are elevated, mainly in the early stages of SAT [11]. Serum TSH, free T4, and T3 should be measured in all patients with clinical suspicion of SAT. It is recommended to check ESR and CRP levels, with or without a radioactive iodine uptake scan. An elevation of ESR and/or CRP levels and a low radioiodine uptake during the hyperthyroid phase help confirm the diagnosis [12]. Thyroid US is a useful tool to assess for any nodules or cystic changes, and the Doppler US is useful to distinguish SAT (decreased blood flow during the hyperthyroid phase) from Graves' disease (increased flow). Patients with disseminated infection and suspicious for a thyroid abscess, it is reasonable to do fine-needle aspiration [13].

The diagnosis of thyroid storm is based upon the presence of severe and life-threatening symptoms (hyperpyrexia T> 104 F°, cardiovascular dysfunction, altered mentation) in a patient with biochemical evidence of hyperthyroidism (elevation of free T4 and/or T3 and suppression of TSH). In 1993, Burch and Wartofsky introduced a scoring system using precise clinical criteria for the identification of thyroid storm that is still in use [14]. The clinical criteria (Table 2) include thermoregulatory dysfunction, cardiovascular dysfunction, central nervous system effects, heart failure, gastrointestinal-hepatic dysfunction, and, finally, a precipitant history. A score of 45 or more is highly suggestive of thyroid storm, whereas a score below 25 makes thyroid storm unlikely. A score of 25 to 44 is suggestive of an impending storm. While this scoring system is likely sensitive, it is not very specific [13]. Our patient scored a total of 95 points (T>104 F° (30 points), lethargy (20 points), nausea/vomiting/abdominal pain (10 points), HR 134 (20 points), pedal edema (5 points), and precipitating event (10 points)), confirming the diagnosis of thyroid storm.

**Table 2:** Diagnostic criteria for thyroid storm: a score of 45 or more is highly suggestive of thyroid storm, a score of 25 to 44 supports the diagnosis, and a score below 25 makes thyroid storm unlikely Adapted from Burch HB and Wartofsky L [14]

Thermoregulatory dysfunction				Cardiovascular dysfunction			
Temperature ( F °)				Tachycardia			
99 to 99.9			5	99 to 109			5
100 to 100.9			10	110 to 119			10
101 to 101.9			15	120 to 129			15
102 to 102.9			20	130 to 139			20
103 to 103.9			25	>= 140			25
> =104			30	Atrial Fibrillation			10
Central nervous system effects				Heart failure			
Mild ( agitation)			10	Mild (Pedal edema)			5
Moderate			20	Moderate (bibasilar rales)			10
Delirium				Severe (Pulmonary edema)			15
Psychosis				Gastrointestinal-hepatic dysfunction			
Severe lethargy				Moderate			10
Severe			30	Diarrhea			
Seizure				Nausea/Vomiting			
Coma				Abdominal pain			
Positive precipitant history 10				Severe (unexplained jaundice)			20

It is recommended to determine the etiology of thyrotoxicosis in patients with thyroid storm by measuring thyrotropin receptor antibodies (TRAb) or thyroid-stimulating antibodies (TSI), with thyroid ultrasound (US) or radioiodine uptake. However, it should not delay the prompt treatment of patients with clinical manifestations of thyroid storm. Our patient had a clinical, laboratory, and imaging findings of SAT as is the case with thyroid storm [15].

The management of thyroid storm should be in an ICU since the mortality is substantial [16]. Generally, the management of thyroid storm or impending storm is the same and typically consists of multiple medications, each of which has a different mechanism of action. Beta-blockers are used to control the symptoms and signs induced by an increased adrenergic tone. Thionamides are used to block new thyroid hormone synthesis, and the iodine solution is used to block the release of thyroid hormone. Glucocorticoids reduce T4-to-T3 conversion, promote vasomotor stability, and possibly treat an associated relative adrenal insufficiency from stress. Bile acid sequestrants can be used in severe cases to decrease enterohepatic recycling of thyroid hormones [16]. Timely recognition, supportive care, and treatment of any precipitating factors (eg, infection) along with specific therapy directed against the thyroid, are critical for the best clinical outcome. Many patients require substantial amounts of fluid while others may require diuresis because of congestive heart failure. Hyperpyrexia should be aggressively managed, and preferably acetaminophen is used instead of aspirin since the latter can increase serum free T4 and T3 concentrations by interfering with their protein binding. In addition, cold intravenous fluids and cooling blankets may be used if the temperature exceeds 104 F° [16-17].

Propranolol is the beta-blocker of choice because, in high doses, it inhibits type 1 deiodinase, which may help reduce serum T3 levels. Therefore, it should be used with a dose to achieve adequate control of heart rate, typically 60 to 80 mg orally every four to six hours. Propranolol requirements may be quite high because of increased drug metabolism as a result of hyperthyroidism. Although thionamides such as propylthiouracil (PTU) or methimazole are generally used in thyroid storm, their utilization in a thyroiditis-induced storm is controversial. If used, PTU is favored over methimazole because of PTU's effect on decreasing T4-to-T3 conversion. In our patient, we used PTU 150 mg every eight hours and monitored the level of T4 daily, which trended down slowly over five days ( >8, >8, 6.46, 4.75, 3.48, 2.88, 1.58, 1.33). Except for a thyroiditis-induced storm, iodinated solutions can be used and should be delayed for at least one hour after thionamides to prevent the iodine from being used as a substrate for new hormone synthesis. In addition, we also administer glucocorticoids such as hydrocortisone 100 mg intravenously every eight hours. Cholestyramine (4 g orally four times daily) may also be of benefit in severe cases to reduce enterohepatic recycling of thyroid hormones [18].

In thyroid storm secondary to Grave's disease or toxic nodules, patients can sometimes develop thionamides-related agranulocytosis or hepatotoxicity. In these cases, thyroidectomy is the treatment of choice. If thyroidectomy is indicated, preoperative treatment with beta-blockers, corticosteroids, iodinated solutions, and cholestyramine should be used for five to seven days (and not more than 10 days) to avoid the Wolff-Chaikoff effect [18]. Plasmapheresis is an investigational therapy in thyroid storm, as it removes cytokines, antibodies, and thyroid hormones from plasma [19]. After clinical improvement of the central nervous system and cardiovascular manifestations, glucocorticoids can be tapered and discontinued. B-blockers can be withdrawn, but only after thyroid function tests have returned to normal. If used, PTU should be titrated to maintain euthyroidism and then discontinued [20].

## Conclusions

Subacute thyroiditis is a self-limited inflammatory disease of the thyroid gland that rarely causes a life-threatening thyroid storm. It is a clinical diagnosis, and the presence of neck pain and tender diffuse goiter is sufficient to establish the diagnosis. Serum TSH, free T4, and T3 should be measured in all patients with clinical suspicion of SAT. The standard treatment of thyroid storm is supportive care, corticosteroids, and beta-blockers. Although PTU utilization in a thyroiditis-induced storm is controversial, it should be considered in severe cases.
